# Discovery of common sequences absent in the human reference genome using pooled samples from next generation sequencing

**DOI:** 10.1186/1471-2164-15-685

**Published:** 2014-08-16

**Authors:** Yu Liu, Mehmet Koyutürk, Sean Maxwell, Min Xiang, Martina Veigl, Richard S Cooper, Bamidele O Tayo, Li Li, Thomas LaFramboise, Zhenghe Wang, Xiaofeng Zhu, Mark R Chance

**Affiliations:** Center for Proteomics and Bioinformatics, Case Western Reserve University, Cleveland, OH USA; Department of Electrical Engineering & Computer Science, Case Western Reserve University, Cleveland, OH USA; Case Comprehensive Cancer Center, Case Western Reserve University, Cleveland, OH USA; Department of Genetics and Genome Science, Case Western Reserve University, Cleveland, OH USA; Department of Family Medicine and Community Health, Case Western Reserve University, Cleveland, OH USA; Department of General Medical Sciences, Case Western Reserve University, Cleveland, OH USA; Department of Epidemiology and Biostatistics, Case Western Reserve University, Cleveland, OH USA; Department of Pharmacy, Suzhou Health College, Suzhou, Jiangsu 215009 P. R. China; Department of Public Health Sciences, Stritch School of Medicine, Loyola University Chicago, Maywood, IL USA

**Keywords:** Missing common sequence, De novo assembling, Next generation sequencing, Expression in brain, Transcription factor binding, Genome evolution

## Abstract

**Background:**

Sequences up to several megabases in length have been found to be present in individual genomes but absent in the human reference genome. These sequences may be common in populations, and their absence in the reference genome may indicate rare variants in the genomes of individuals who served as donors for the human genome project. As the reference genome is used in probe design for microarray technology and mapping short reads in next generation sequencing (NGS), this missing sequence could be a source of bias in functional genomic studies and variant analysis. One End Anchor (OEA) and/or orphan reads from paired-end sequencing have been used to identify novel sequences that are absent in reference genome. However, there is no study to investigate the distribution, evolution and functionality of those sequences in human populations.

**Results:**

To systematically identify and study the missing common sequences (micSeqs), we extended the previous method by pooling OEA reads from large number of individuals and applying strict filtering methods to remove false sequences. The pipeline was applied to data from phase 1 of the 1000 Genomes Project. We identified 309 micSeqs that are present in at least 1% of the human population, but absent in the reference genome. We confirmed 76% of these 309 micSeqs by comparison to other primate genomes, individual human genomes, and gene expression data. Furthermore, we randomly selected fifteen micSeqs and confirmed their presence using PCR validation in 38 additional individuals. Functional analysis using published RNA-seq and ChIP-seq data showed that eleven micSeqs are highly expressed in human brain and three micSeqs contain transcription factor (TF) binding regions, suggesting they are functional elements. In addition, the identified micSeqs are absent in non-primates and show dynamic acquisition during primate evolution culminating with most micSeqs being present in Africans, suggesting some micSeqs may be important sources of human diversity.

**Conclusions:**

76% of micSeqs were confirmed by a comparative genomics approach. Fourteen micSeqs are expressed in human brain or contain TF binding regions. Some micSeqs are primate-specific, conserved and may play a role in the evolution of primates.

**Electronic supplementary material:**

The online version of this article (doi:10.1186/1471-2164-15-685) contains supplementary material, which is available to authorized users.

## Background

The identification and characterization of all common sequence variants in the human genome has transformed our understanding of segregating diversity, population genetics, and disease susceptibility. SNPs have traditionally been thought to be the dominant source of sequence variation although other sequence variants, such as sequence duplication and deletion recognized microscopically [1], were described decades ago. Significant efforts have been made to document all common SNPs in the human genome using traditional technologies [2, 3]. In the past decade, the completion of the human reference genome and development of high throughput technologies, such as microarray and next generation sequencing (NGS), have revolutionized methods for the efficient assessment of genome composition in human populations. More than 35 million SNPs have been documented based on the analysis of data from the latest technologies [4–8]. However, genome surveys also revealed an unexpectedly large extent of other categories of sequence variants, including copy-number variants (CNV), inversions, translocations, and small to large sequence insertions and deletions [7].

The human reference genome played a significant role in the detection of sequence variants, because it was extensively used for probe design and array generation and mapping short reads in NGS. Given that we now know that sequence insertions and deletions are common, and considering that around 80% of the reference genome is derived from a single individual [9, 10], it is reasonable to expect that many common sequences, i.e. those present in at least 1% of the population, may be absent in the reference data due to missing copies in the few individuals that were studied. Recently, multiple studies, using both NGS and traditional capillary sequencing, have reported novel sequences that are absent in the reference genome, but are present in at least a few individuals [11–14]. For example, using capillary sequencing technology, a recent study identified more than 2,363 novel sequences (in total, more than 2 Mb) in the genomes of nine individuals [11]. Many of these appear in more than one individual, implying that these sequences may be common in human populations. Furthermore, *de novo* assembly of several individual genomes that were deeply sequenced using NGS also revealed novel sequences [12, 13]. Based on these findings, the amount of novel sequences that are not present in the reference genome was estimated to be around 20–40 Mb for each individual [12]. Comprehensive discovery of all common sequences that are absent in the reference genome would require sequencing of a large number of individual genomes that are representative of the human population. Currently, neither capillary sequencing nor deep sequencing using NGS are suitable for these purposes, since they require either intensive labor or the cost is still prohibitive for deep sequencing of a large number of individuals.

We have developed a method to identify novel sequences in individuals using One End Anchor (OEA) reads from paired-end sequencing where one end of the pair can be uniquely mapped to the reference genome (termed “anchor read”) while the other cannot be mapped at all (termed “orphan read”) [11]. A similar method was developed for the detection of virus sequences [15]. We extend this strategy by pooling shallow sequencing data (e.g., with 4× coverage) from a large number of individuals to systematically identify sequences common in the human population that are absent in the reference genome. For this purpose, we applied this method on genomic data from hundreds of individuals generated from Phase 1 of the 1000 Genomes Project [7]. Our pipeline identifies micSeqs by pooling orphan reads from 363 individuals of African, Asian, and European origin followed by *de novo* assembly of these orphan reads to generate candidate micSeqs. We used various strategies to filter out false micSeqs, and systematically validated the remaining micSeqs through comparative genomic analysis. Direct experimental examination of randomly selected fifteen micSeqs on 38 additional samples confirmed that all fifteen micSeqs were present in at least one individual. Additional analysis suggested many micSeqs are functional genomic elements involved in biological functions.

## Results

### Identification of micSeq by pooling samples from 1 K Genome Project

**Figure 1:**
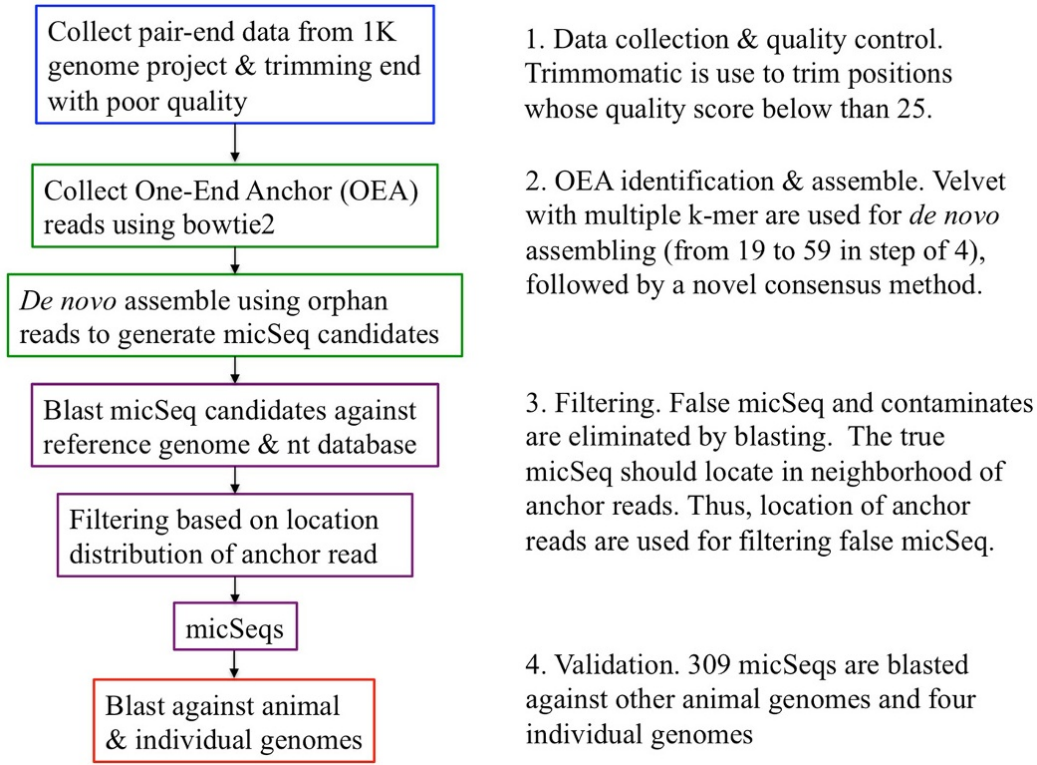
**Summary of pipeline for discovery of micSeqs.** The pipeline has four steps: 1) data collection and quality control; 2) OEA identification and *de novo* assembly; 3) filtering false micSeqs; and 4) validation by comparative genomics.

**Figure 2:**
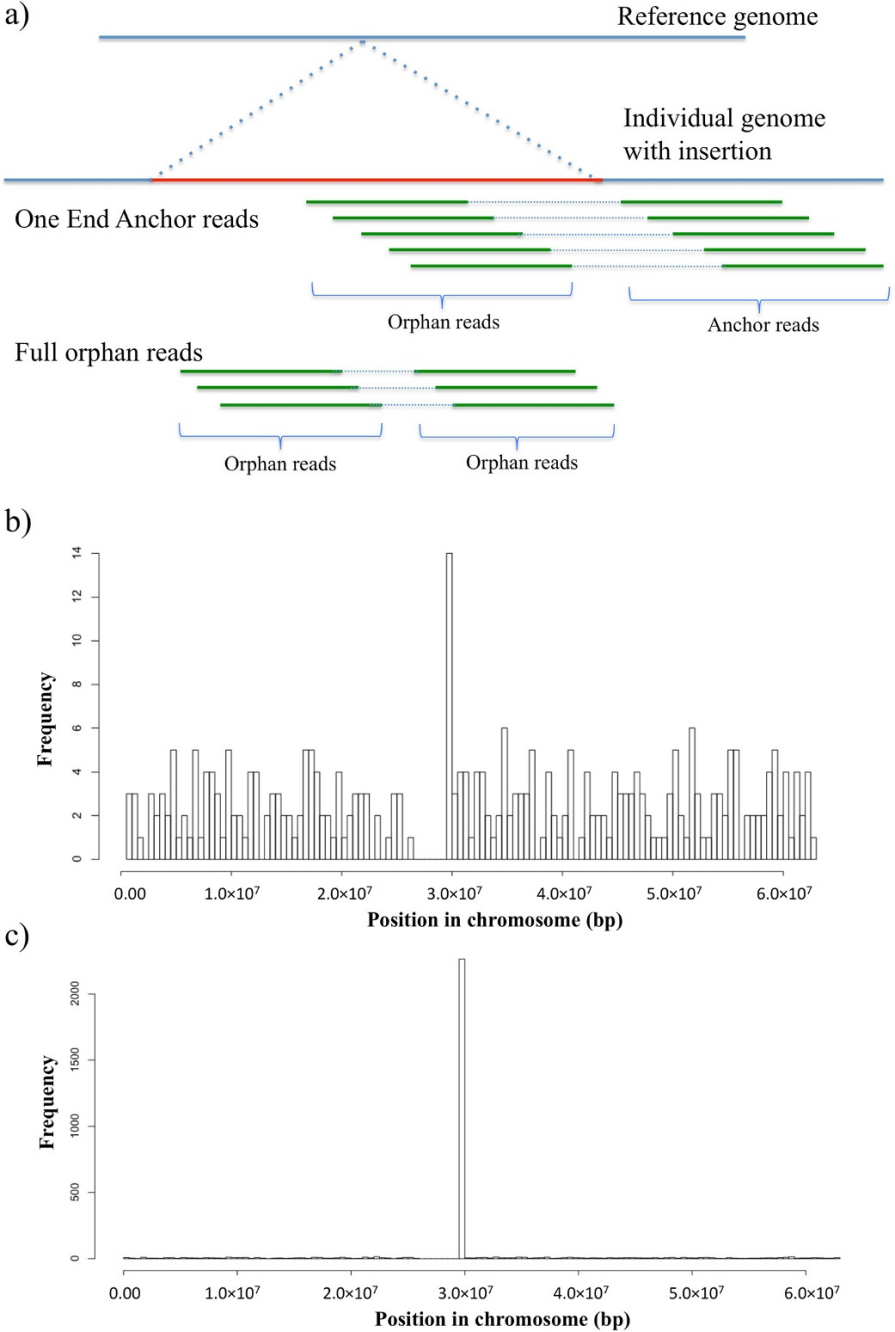
**One End Anchor reads.** Due to the limited insert size, orphan reads are in the neighborhood of anchor reads in paired end sequencing, thus, the location of anchor reads can be used to filter out the false sequences and estimate the location of true sequences. Note that full orphan reads could be used to help detect longer insertion sequences. **a)** Illustration of OEA reads. **b)** the anchor reads corresponding to one micSeq candidate are distributed along the chromosome with very modest coherence in one location, it is very likely this is a false micSeq. Candidate micSqs of this type (>50% of reads distributed across the chromosome) are filtered out; **c)** the anchor reads are highly clustered in one short region, these are brought forward for validation analysis.

Our pipeline to detect micSeqs, contains four steps (summarized in Figure 1): 1) Data collection and quality control; 2) Identification of One End Anchor reads (Figure 2) and *de novo* assembly of micSeqs using pooled OEA reads from 363 individuals. Pooling permits the use of shallow sequence data and the identification of common sequences is dependent on a stringent coverage cutoff for *de novo* assembly, in this case at least 50× coverage; 3) Identification and filtering of false micSeqs based on the genomic location of anchor reads corresponding to the orphan reads that are assembled together (Figure 2); 4) Validation of filtered micSeqs *via* comparative genomic analysis on a database of vertebrate genomes, comparison to the deeply sequenced genomes of four individuals, and comparison to RNA-Seq data, as well as direct experimental examination of micSeqs.

**Figure 3:**
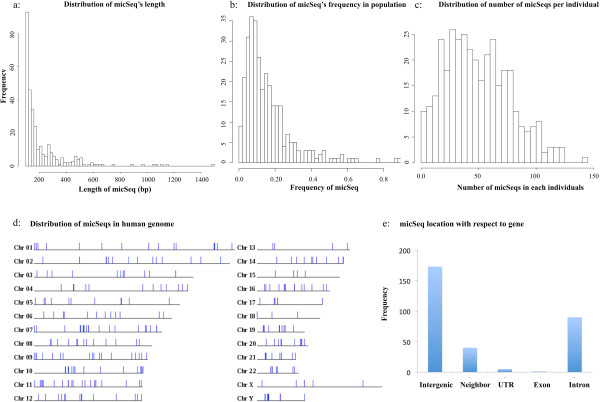
**Characteristics of micSeqs. a)** the length distribution of 309 micSeqs, the average length of 309 micSeqs is 221 bp; **b)** the distribution of micSeq frequency in the human sample represented in the 1 K Genome project data, the individuals are considered to contain the micSeq if they contribute at least three orphan reads; 70% of micSeqs have higher than 5% frequency, i.e. present in at least 18 individuals; **c)** the distribution of number of micSeqs in each individual, on average each individual has 50 micSeqs; **d)** the distribution of micSeqs along human genome; **e)** the distribution of micSeq location with respect to RefSeq genes.

The pipeline was applied to paired-end sequencing data for 363 individuals of African (YRI, 88 individuals), Asian (CHB, 88, and JPT, 85) and European (CEU, 102) origin obtained from the Phase 1 release of the 1 K Genomes Project. We identified 309 micSeqs with a length of at least 100 bp. In total, the 309 micSeqs contained 68,424 bp (micSeq sequences along with predicted locations, neighboring genes, supporting evidence and other information are available in http://proteomics.case.edu/micseqdb/). The longest micSeq had 1,494 bp and the mean length is 221 bp (Figure 3a). Over 70% of individual micSeqs are present in at least 5% of the population studied (≥18 individuals, Figure 3b), and on average each individual has 50 micSeqs (Figure 3c) comprised of 5Kb or more sequences that are absent in reference genome. We investigated the location of micSeqs in chromosomes based on their anchor position, and found that these micSeqs are relatively uniformly distributed across the genome (Figure 3d).

Previous methods have used One End Anchor (OEA) and orphan reads to identify novel sequences present in individuals using deep sequenced genomes [11, 14]. In contrast to these studies that focus on individual genomes, we here focus on the population by deriving strength from pooled reads, i.e., our objective is to identify sequences that are novel and *relatively common in human populations*. Currently, the number of deep-sequenced genomes is still limited. To take advantage of availability of large number of shallow-sequenced genomes, we pooled OEA reads from different individuals to identify micSeqs. The pooled nature of the reads we utilized also requires methodological improvements as compared to existing methods. Namely, since the number of pooled reads is very large, our method utilizes the position of the corresponding anchor reads to improve computational efficiency by filtering out false sequences (Figure 2). The location information is also valuable for further annotation and validation using Sanger sequencing, for example, for primer design. Finally, our results further contribute to the literature by validating and investigating the conservation and evolution of the identified sequences using comparative genomics approach, as described below.

### Validation of micSeq by comparative genomics and Sanger sequencing on additional samples

**Figure 4:**
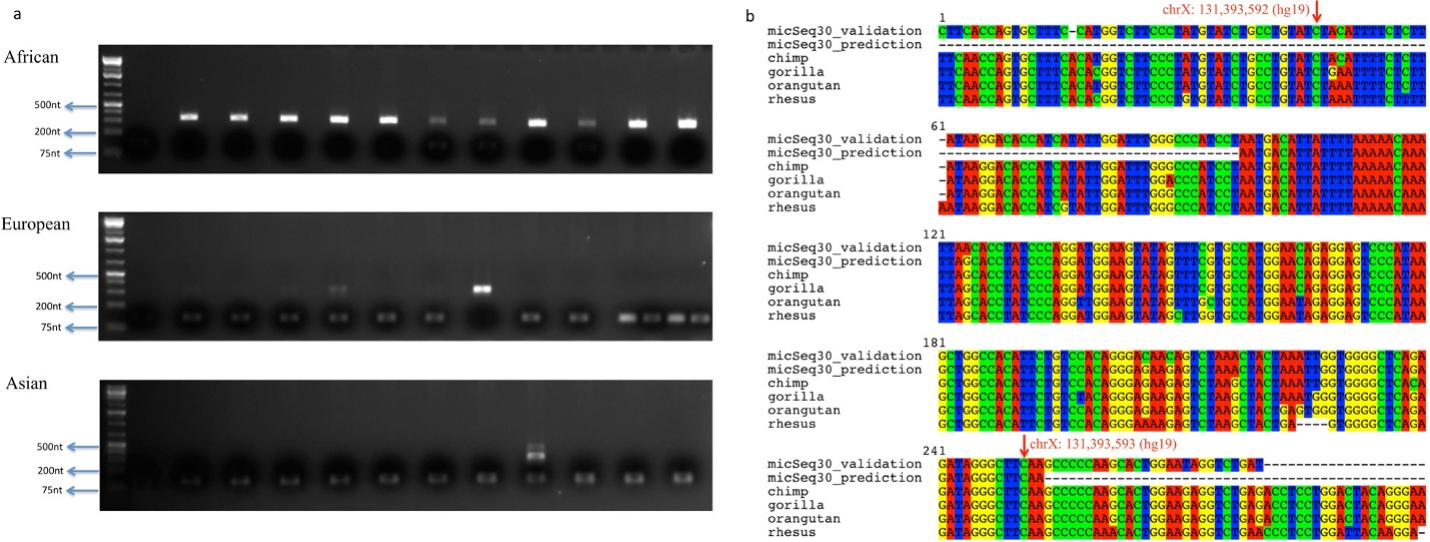
**PCR of individuals and evolutionary conservation of micSeq30. a)** PCR results for micSeq30 for samples from three populations used for validation. The allele frequency clearly shows population stratification for this micSeq: with higher frequency in African, and much lower in others, p-value < 10^−5^ for African *vs* non-African (Table 1). **b)** Sanger sequencing results for PCR amplified DNA, predicted sequence of micSeq30, and homologs from other primates. The genomic coordinates for other primates are: chimp: chrX: 132, 861, 450-132, 861, 750; gorilla: chrX: 129, 774, 750-129, 775, 050; orangutan: chrX: 131, 684, 602-131, 684,902; and rhesus: chrX: 130, 465, 777-130, 466, 071. The red arrows show the corresponding coordinates in human reference genome (hg19). The inserted position of micSeq30 in the reference genome is: chrX: 131, 393, 592, and, the corresponding anchor reads for micSeq30 locate in the region of chrX: 131, 393, 154-131, 393, 947.

Comparison of micSeqs with genome sequences from additional individuals provides validation for many micSeqs, while comparison with genomes from other vertebrate species can evaluate whether they are conserved during evolution. We compared the micSeqs with genome sequences from four individuals (two European, one Chinese, one African), particularly focusing on the sequences that cannot be mapped to the reference [12, 16, 17]. Three micSeqs (with 99% sequence similarity) are present in all four individuals, while 118 micSeqs were found to be present in at least one of the four. More micSeqs (80) were detected in J. Craig Venter’s genome compared to other individual genomes (32 micSeqs for the African individual, 22 for James Watson, and 25 for the Chinese individual). Venter’s genome was sequenced using capillary sequencing [16], previous studies have indicated that novel sequences were often partially sequenced or were missing altogether in genomes sequenced using NGS technologies [11]. Seven of the micSeqs had high sequence similarity with novel insertions previously detected in nine individuals [11]. This modest overlap suggests that the previous study identified many novel sequences that are more rare than those identified here. When compared with genomes from other vertebrates, none of the micSeqs had homologs in genomes other than those of primates (viz, chimpanzee, gorilla, orangutan, gibbon, rhesus, and marmoset). We were able to find potential homologs for 45% of micSeqs (139 micSeqs, Table 1) in at least one primate with sequence similarity higher than 90%. 106 micSeqs had homologs in at least three primates, indicating that they are conserved during evolution and may have functional roles. Data for micSeq30, including the homologs in primates, the population distribution, predicted and validated sequences (see below), are shown in Figure 4. The conservation of micSeqs in multiple primate species provides an opportunity to locate their exact position in the human genome at the resolution of single nucleotide (Figure 4). For micSeqs without homologs in other species, the location of corresponding anchor reads can provide reliable position information with 1Kb resolution (Figure 2a). The estimated locations can be found at the online database: http://proteomics.case.edu/micseqdb.

**Table 1:** **Number of micSeq homologs in other primates**

Primates	Chimp (panTro3)	Gorilla (gorGor3)	Orangutan (panAbe2)	Gibbon (nomLeu)	Rhesus (rheMac2)	Marmoset (calJac3)
Homologs	126	102	103	92	59	15

None of 309 micSeqs has homolog other than primates. In contrast, 139 micSeqs have at least one homolog in other primates. Note that the number of homolog in one primate agrees with the distance to human, i.e., more homolog present in species close to human.

Among 139 micSeqs conserved in primates, fifteen micSeqs were randomly selected for experimental validation using PCR on an additional 38 samples from individuals with African (12 samples), Asian (12 samples) and European (14 samples) ancestry. The primers used can be found in (Additional file 1: Table S1) along with the targeted micSeqs. The PCR and sequences for micSeq30 were shown in Figure 4, which clearly shows the presence of micSeq30 in most of African individuals while it is absent in the other populations. Furthermore, the DNA sequences from Sanger sequencing confirms our prediction for micSeq30 in terms of both its sequence and position. We also selected a few bands that don’t contain micSeqs (the lower bands) for Sanger sequencing to verify the absence of micSeqs. One example was included in (Additional file 1: Figure S1 and S2). All 15 micSeqs were detected in at least one individual, i.e., bands of appropriate size are observed in DNA blots (Table 2). Nine out of 15 micSeqs showed extensive population stratification with a significantly higher allele frequency in African *vs.* non-African individuals, consistent with previous studies [11]. The micSeq frequencies in different populations estimated from 1 K genome data follow similar trends.

**Table 2:** **Frequency of micSeqs from validation samples**

micSeqID	Freq from validation data of Europe samples	Freq from validation data of Asian samples	Freq from validation data of Africa samples	P-value for African ***vs***non-African
micSeq11220	0.46	0.54	*0.63*	0.33
micSeq13298	0.11	0.08	*0.29*	**0.043**
micSeq16601	0.07	0	*0.13*	0.31
micSeq18764	0.11	0.08	*0.38*	**0.0021**
micSeq11710	0	0.25	*0.33*	**0.0030**
micSeq143	0	*0.92*	0.33	0.61
micSeq1824	0.14	*0.29*	0.13	0.53
micSeq2373	0	0.08	*0.33*	**0.0010**
micSeq2382	0.32	0.42	*0.50*	0.31
micSeq30	0.25	0.04	*0.79*	**0**
micSeq71	0.29	0.29	*0.58*	**0.022**
micSeq634	*0.14*	0	0	0.30
micSeq9196	0.29	0.29	*0.92*	**0**
micSeq349	0	0.58	*0.75*	**0.00013**
micSeq28	0.54	0.13	*0.67*	**0.013**

The sample sizes are 14 for the European group, 12 for the African group, and 12 for the Asian group. For each micSeq, frequency in italic font indicates the maximum frequency. 12 out of 15 micSeqs show higher frequency in the African population than the other populations; 9 of them are statistically significant at the 5% level as indicated by bold font.

**Figure 5:**
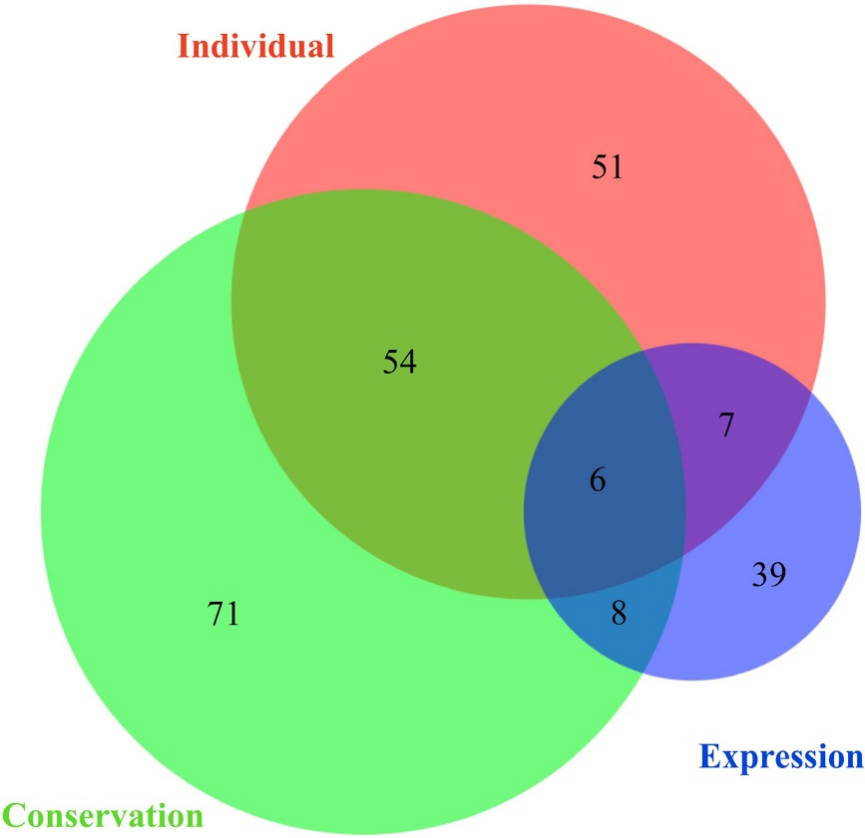
**Venn diagram of micSeq validation analyses.** The overlap of micSeqs observed be homologous to primate sequences, and in high coverage data from four individual genomes, and in mRNA expression data of human brain samples. In total, 236 micSeqs or 76% have independent evidence for their presence in human genome sequences.

In summary, 236 micSeqs out of 309 that were identified (76%) were supported by independent evidence for their presence in humans. The independent evidence included detection in individual genomes, existence of homologs in other primate genomes, or support from expression arrays in human brain tissue (see below), as summarized in Figure 5. All of 15 selected micSeqs have been confirmed using PCR experiments in 38 additional individuals from Africa, Asia and Europe.

### Some micSeqs are highly expressed in human brain

Potential gene associations of micSeqs were established by comparing their genomic locations derived from the Anchor Reads with those of genes obtained from RefSeq database. The results are summarized in Figure 1e; 214 micSeqs locate in intergenic region (among them, 40 micSeqs located in the 5 Kb region of either side of annotated genes) while 95 are seen in the UTR or intron regions of RefSeq genes. Only one micSeq locates in the coding region (micSeq2153 in exon of MUC6). The RefSeq genes associated with micSeq can be found in the online database (see: http://proteomics.case.edu/micseqdb/).

**Figure 6:**
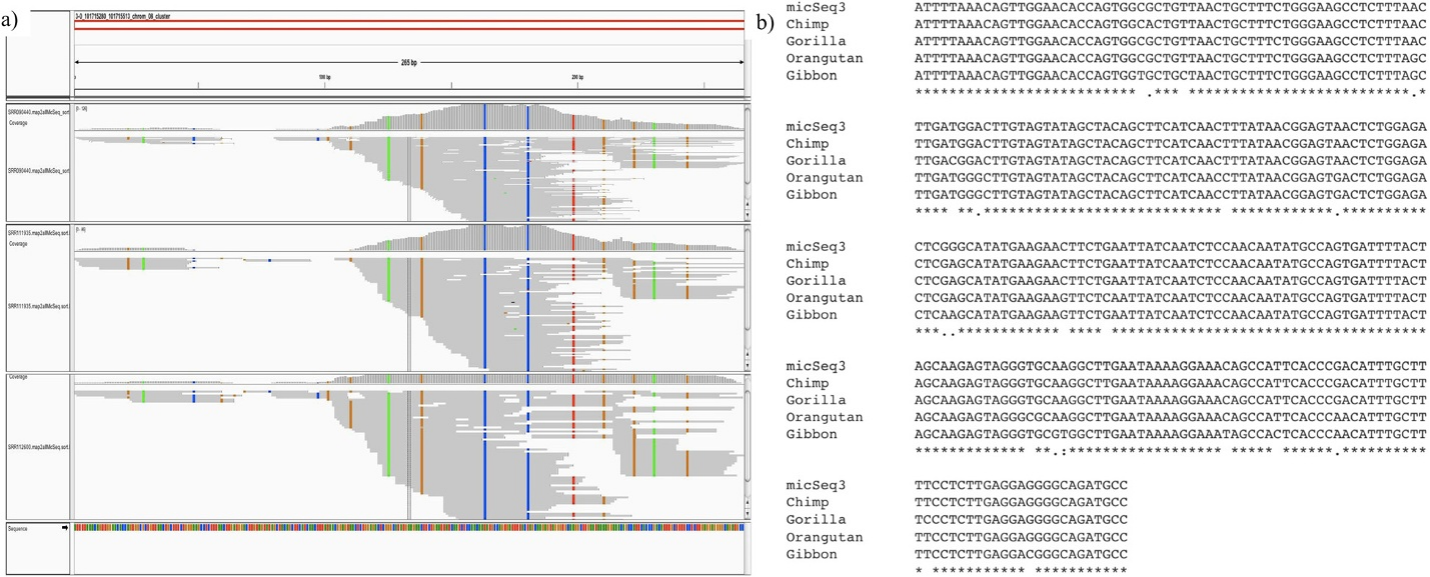
**Example of expressed micSeqs detected from RNA-seq data. a)** Reads aligned to micSeq3. Note that the reads depth can be up to more than 100, indicting it is moderately expressed. The color columns indicate that the reads have different nucleotides with micSeq3 probably because of SNPs or RNA editing. **b)** Homologs of micSeqs with high sequence similarity can be identified in other primates.

The complete transcriptome of the human brain contributed to our understanding of the development and function of different brain regions and functional circuits. We further investigated whether the identified micSeqs were expressed in the brain. For this purpose, we collected and analyzed 59 RNA-seq data sets of samples from various brain regions [18–24]. 11 micSeqs have more than 100 reads in individual samples, suggesting that they are relatively highly expressed. For example, in each of six samples (SRR090440, SRR111935, SRR112600, ERR030890, SRR111936 and SRR309138), there are more than 100 reads mapped to micSeq3, three of which are shown in Figure 6a. Note that several positions show differences between assembled reads with micSeq3; the reason might be SNP or RNA editing since editing events are prevalent in the brain [25]. The read depths were up to 125 in one sample (SRR090440). We further identified one EST sequence from brain in dbEST (BF687531.1) that has significant high similarity with micSeq3 (the sequence alignment can be found in Additional file 1: Figure S3), which further confirms the existence and expression of micSeq3. MicSeq3 has a length of 265 nt, and is located in the 3′UTR of gene PABPC1 (chr8:101,715,280-101,715,513), which codes a poly (A) binding protein. The rate of expression of this micSeq in some individuals suggests that it might have a regulatory function or help to code an alternative exon for PABPC1. Moreover, micSeq3 has homologous sequences with high sequence similarity identified in four other primates: chimpanzee (sequence similarity, 99.2%), gorilla (98.9%), orangutan (96.6%) and gibbon (94.3%), providing further evidence for its importance (Figure 6b). However, further detailed analysis are needed to determine the exact function: e.g. if they are missing exons or noncoding RNAs, and if these micSeqs are expressed in other tissues or are brain specific.

### Three micSeqs containing TF-binding regions

**Figure 7:**
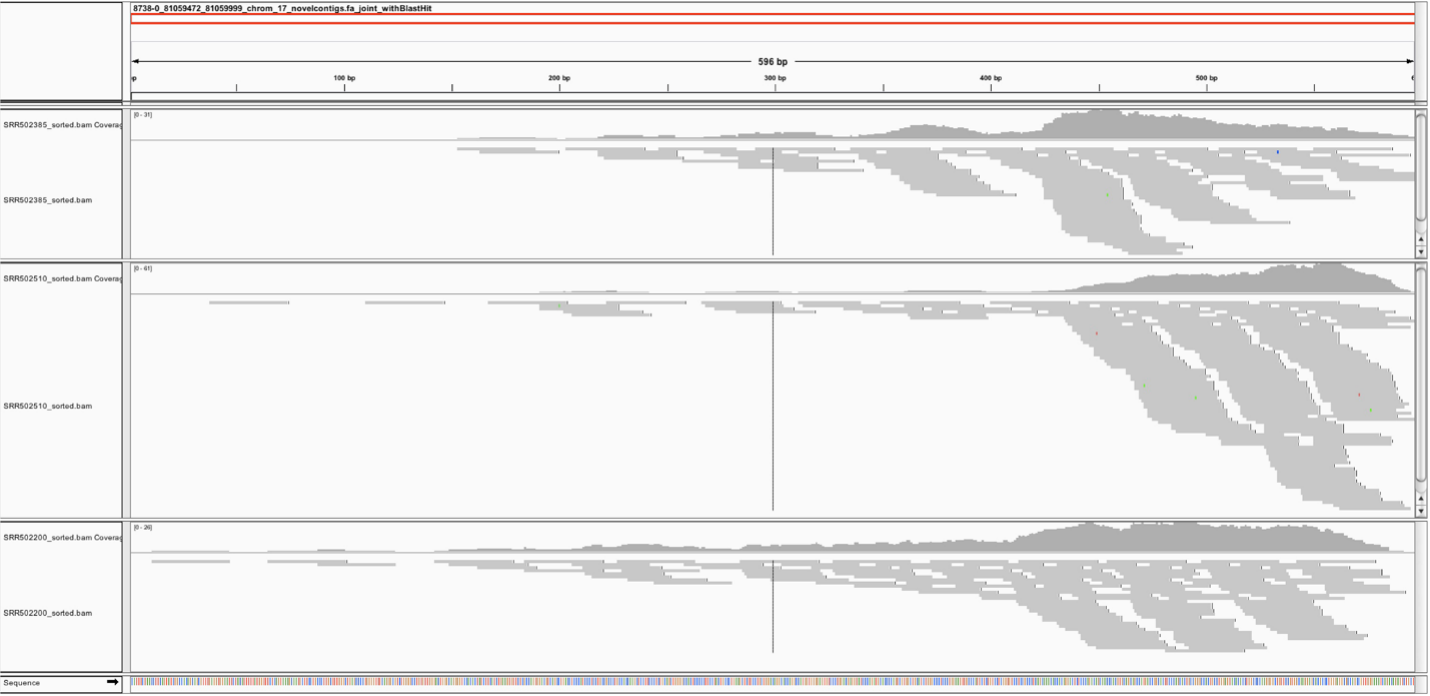
**TF binding region in micSeq8738.** Reads from ChIP-seq can be mapped to part of micSeq8738, indicating micSeq8738 contains TF binding region. Reads from three ChIP-seq experiments are showed in this figure. Note that micSeq8738 might have only part of TF binding region due to the high cutoff threshold (50 coverage).

Recently, the ENCODE project published a series of comprehensive studies to uncover the functional elements in the human genome, including analysis of ChIP-seq data for 201 transcription factors (TF) in 147 cell types [26, 27]. To investigate if the identified micSeqs contain TF-binding regions we analyzed the whole ChIP-seq data set from the ENCODE project (~1900 datasets). Our analysis provided strong evidence that three micSeqs (micSeq8738, micSeq292, and micSeq9698) are likely to bind to TFs, such as POL2, STAT3, FOXA1. The detailed results can be found in Supplemental Material including the number of reads mapped to micSeqs, the TFs and SRA ids. Figure 7 shows that more than 100 reads from three ChIP-seq datasets were mapped to micSeq8738, and the highest coverage was 61×. However, as mentioned above, due to the stringent criteria to assemble micSeqs (at least 50×) and lower coverage of NGS data from the 1 K genome project, most of micSeqs are short (average of 221 nt), and are likely to be only part of longer sequences in humans. The ChIP-seq reads cluster in the end of micSeq8738 (Figure 7), suggesting the possibility that micSeq8738 contains only part of the TF-binding region.

Due to potential concerns of sequencing bias in ChIP-seq experiments, comparison with appropriate control data (ChIP-seq experiments using so-called “input DNA”, non-ChIP genomic DNA) is critical to filter out false binding sites. For each of micSeq8738, micSeq292, and micSeq9698, there were very limited reads from control experiments that could be mapped to them - less than 10 reads in the vast majority of samples (see Additional file 2). For example, in the case of micSeq8738, on average, 72 reads map to ChIP-seq experiments for 15 TFs on HeLa-S3 cells, compared with only 7 reads to the 14 control samples. Both micSeq8738 and micSeq292 are located in the neighborhood of noncoding RNAs; micSeq8738 is 1.5 kb away from one lincRNA, AC144831.1, while miRNA1268A is very close to micSeq292 (1 kb away). These micSeqs might regulate expression of these noncoding RNAs through TF-binding. Moreover, these micSeqs occur at a high frequency in the population; more than half of 363 individuals contribute at least one read to assemble them, and one third contribute at least two reads, implying that their deletion in the reference genome could be considered a rare variant.

### Comparison with the latest reference genome

In December 24, 2013, the Genome Reference Consortium announced the public release of GRCh38, the latest version of the human reference genome assembly, and phase 2 of 1000 Genomes Project used a combination of reference GRCh37 (hg19) and unlocalized and unplaced contigs as reference genome. We searched our sequences against these two latest versions of the reference genome for micSeqs. Sequences with more than 95% similarity were detected for 43 micSeqs for GRCh38 and 80 for the reference used by the 1000 Genomes Project, which further validates our approach. These results also suggest that the remaining micSeqs are good candidates for inclusion in future releases of the reference human genome.

## Discussion

A comprehensive catalogue of genetic variants, including SNPs, indels, CNVs, and common and rare variants is an essential resource for researchers attempting to identify variants that affect phenotypic traits, complex genetic diseases, and responses to drugs and environmental factors. Several large-scale projects, such as HapMap and 1 K Genome Project [6, 7], have made significant progress toward documentation of all common variants in several human populations, and laid the basis for the success of current generation of genome wide association studies (GWAS) [28]. Although the ‘common variant- common diseases’ hypothesis was not confirmed by GWAS [29, 30], and the basis of the so-called ‘missing heritability’ problem is still not well understood [31, 32], many common variants with small effects and many rare variants with large effects need to be identified and investigated [30].

MicSeq is a special form of an indel, in that these sequences are absent in the reference genome. Detecting intermediate length to large indels (from 20 nt to several kb) is challenging with the current high-throughput technologies, and sequences absent in the reference genome are not targeted in virtually all genotyping projects [33]. *De novo* assembly of individual genomes followed by comparison with the reference sequence was a key method used to discover such sequences. As noted, previous analyses of individual genomes deeply sequenced using NGS have identified nucleotide patterns present in specific individuals but absent in the reference genome [12–14, 16, 17]. However, this approach is still prohibitive as a method to identify common variants due to the high cost for deep sequencing of many individuals and assembling a large number of short reads. Our approach takes advantage of One End Anchor reads from paired end reads, and assembles micSeqs by pooling orphan reads of lower coverage NGS data (4× on average) from a large number of individuals. Validation by comparative genomics and PCR experiments on additional samples demonstrate that our method is accurate and effective in detecting micSeqs. However, the high-coverage threshold used in the *de novo* assembly of step 2 (minimum 50×) limits the detected micSeqs to commonly observed sequences instead of rare ones present in only a few individuals. This cutoff could be adjusted based on the number of individuals and the depth of sequencing to identify fewer highly common variants or a greater number of rare variants. It should also be noted that the resulting micSeqs assembled by our method could be part of the true consensus sequence, and a large number of micSeqs in the population remain to be discovered, due to the relatively low coverage of NGS data and only 363 individuals from three populations used in this study.

The origin and evolution of micSeqs in humans can be estimated using a parsimony approach along with the time point of deletion in the ancestors of a sub-population and/or identification of periods that new sequences emerged. None of the 309 micSeqs reported here have homologs beyond primates, indicating that they are relatively young in the human genome and common ancestors of primates first acquired those sequences in their genomes. It is also interesting to note that the number of micSeqs in each primate agrees precisely with the evolutionary distance with humans (Table 1), suggesting that micSeqs were acquired gradually over long time intervals. Possible mechanisms to acquire new sequences include duplication followed by dramatic changes, admixture with other closely related species, and horizontal sequence transfer from other species. Duplication is a major evolutionary event occurring across the genome sequence, and contributes significantly to shape and refactor the functionalities of the organism [34]. However, duplication may not be a mechanism relevant to micSeqs since no paralogous sequences can be detected in human and other primates. Horizontal DNA sequence transfer, particularly through retroviral infection [35], is the process by which an organism incorporates DNA sequence from evolutionarily unrelated organism. This phenomenon is well documented in evolution of bacterial and parasitic unicellular eukaryotes [36]. Recent studies show strong evidence of transfer of DNA to higher eukaryotes, such as *Drosophila*, wheel animals and even rodents [37–40]. However, it is still not clear if horizontal transfer can occur in primates. Recent analyses of Neandertal and archaic hominid genomes show that up to 6% percent of their genomes can contribute to genomes of one human population [41–43], suggesting at least two admixture events in the course of human evolution. Thus, the micSeqs possibly resulted from the admixture of other closely related extinct species.

In our data 253 micSeqs (82%) have either homologs in other primates or are present in African populations. Thus, absence of the majority of micSeqs in part of the global human population are due to deletions in recent human evolution, since strong evidence supports the origin of modern humans occurred in a relatively restricted geographic distribution in East Africa. Thirty three of 253 have homologs in other primates, but are absent in Africans, suggesting in a complementary fashion that they were deleted in African populations. Fifty six micSeqs have no homolog in primates and are absent in African populations; one possible mechanism to explain this finding is the acquisition of new sequences by ancestors of contemporary populations after their migration from Africa.

## Conclusions

This work presented here builds on previous several technological and analytic advances, beginning with NGS data or Sanger sequencing from cloned DNA, to identify novel sequence variants in individual genomes [11, 12, 14, 17, 44]. The unique features of this study that have relevance for other data sets include: pooling of orphan reads from many individuals’ NGS data to identify novel common sequences, where the frequency with which the sequence is identified depends on the number of individuals pooled and the depth of sequencing for each individual; and examination of the concordance statistics of the mapping locations provided by the anchor end of the OEA reads to filter out false micSeqs across the population of individuals and/or reads (Figure 2). Clearly this approach could be an effective means of identifying insertions of novel sequences related to human disease by examining pooled NGS data from affected individuals, including tumors or other tissue-specific genomes. However, due to limitations in the assembly of short reads, we may have identified only parts of longer insertions. Assembling the full orphan reads and linking ends to the existing micSeqs could identify additional sequences. Thus both the orphan reads from OEA and/or full orphan reads could be used to identify novel insertions where techniques such as mapping to primate sequences, examination of individual genomes, comparison to RNA-seq data, or PCR and sequencing are used to validate predicted novel insertions. The identification of over 300 sequences - at least 50 per individual - will be a new focus for future functional analyses.

## Methods

### Data collection

The paired-end sequencing data were downloaded from phase one of the 1 K Genome Project [7]. We collected data for 363 individuals whose origins are European (CEU, 102 samples), African (YRI, 88 samples) and Asian (CHB and JPT, 88 and 85 samples respectively). In total, 12,736 fastq files (5,118 files for Europeans, 3,526 for Africans, and 4,092 for Asians) were collected and processed.

### Trimming low quality positions

Software trimmomatic (version 0.22) was used to trim adapter sequences and nucleotides with low quality from the original data [45]. Trimmomatic performed a sliding window trimming with window size of four nucleotides; regions where the average quality falls below 25 were removed. The whole reads were removed if the remaining length was shorter than 20 nucleotides. The corresponding mate read was also removed if one whole read was filtered.

### Aligning to reference genome

Bowtie2 was applied to map paired-end reads to human reference genome (hg19, including chr1-22, X, Y, M and other 44 contigs) [46]. A pair of reads is said to align “discordantly”, if the alignment of the two reads to the reference does not match paired-end expectations (i.e. the mates aren’t in the expected relative orientation, aren’t within the expected distance range, or one read cannot be mapped to reference (orphan read) while its mate can (anchor read), i.e., One-End Anchor (OEA) reads [11]). The discordant pairs were identified and collected, and the OEA reads were determined. The orphan reads mapped to chr1-22, X, and Y were collected and divided into different groups based on the chromosome location of the anchor reads. The locations of anchor reads were recorded and were further used for filtering false micSeqs and identifying the position of genomic location of the final set of micSeqs (see below, Figure 2).

### *De novo*assembly

Velvet was applied for *de novo* assembly of the orphan reads for each chromosome separately [47]. Velvet uses *de Bruijn* graph to construct the assembled sequences. One important parameter of Velvet is the length of a k-mer, whose setting is critical for the accuracy of the assembly [47]. To improve the efficiency and accuracy of the assembly, the multiple k-mer (from 19 to 59 in steps of 4) assembly option was applied, i.e., multiple assembled sequences were generated using different k-mers.

### Collecting candidate micSeqs

A novel algorithm was designed to compare and select the best assembly from the results of Velvet with multiple k-mers. We expected that some of the assembled sequences from Velvet with different k-mers would have high similarity or be even identical; on the other hand, some sequences might be assembled in only one or a few k-mers. To identify such sequences, the assembled sequences were collected for each chromosome, and searched against themselves using blast. A sequence similarity graph was constructed based on the blast results. If two assembled sequences had high similarity (e-value < 10^−10^), they were connected in the sequence similarity graph. Each connected component in the resulting similarity graph corresponded to a group of highly similar sequences. For each connected component, the longest of the sequences represented by the nodes in the component was considered as a candidate micSeq. The algorithm also identified the sequences without any connections that were assembled with one single k-mer.

The coverage cutoff for *de novo* assembly of micSeqs was set to 50×. However, analysis showed that more than 40 genomic regions have more than one candidate micSeqs. It is possible that those candidates are part of one large true micSeq. To increase the length of micSeqs in these regions, 20× coverage cutoff was applied, and the longest assembled sequence was considered as a candidate micSeq.

### Filtering false micSeqs

Candidate micSeqs were filtered to remove false micSeqs through several steps. First, using blast, the candidate micSeqs were searched against human genome to remove known sequences and low complexity sequences. Second, false micSeqs were identified and filtered based on the distribution of location of corresponding anchor reads. If the candidate was a true micSeq, the location of corresponding anchor reads for the assembled sequences would be clustered in the neighborhood of the anchor point (Figure 2). Thus, the candidates were considered as false and filtered out if 50% corresponding anchor reads didn’t cluster together within a 4 Kb region. Third, sequences corresponding to the location of the anchor reads were downloaded from UCSC genome browser website, candidate micSeqs that are alignable to those sequences are removed. Finally, the candidate micSeqs are searched against the NCBI nucleotide database (nt database) to remove potential contaminants from other species, such as microbes and bacteria. Interestingly, we found that 19 *de novo* assembled sequences of orphan reads with length from 101 nt to 866 nt come from Epstein-Barr virus (EBV) with 100% sequence similarity. This is not surprising as the 1 K Genomes Project used lymphoblastoid cell lines through EBV transformation of B-lymphocytes [7]. Notably, more than 90% individual genomes had evidence for at least one of 19 such sequences, which shows such sequences are widespread in the data released from the 1 K Genomes Project.

### Validating micSeqs by comparative genomics

A comparative genomics approach was applied to validate the identified micSeqs. 61 vertebrate genomes were downloaded from UCSC genome browser, and micSeqs were searched against them to identify potential homologies for micSeqs with default set using blast. In last five years, several human individual genomes have been completely sequenced using either capillary or next generation sequencing, and comparison with reference genome showed up to 40 Mb novel sequences present in those individual genomes [12, 16, 17]. We collected such novel sequences from four published individual genomes, and also compared with micSeqs.

### Joint *vs*population *de novo*assembling

In order to detect population specific micSeqs and micSeqs with lower frequency in each populations, two *de novo* assembly approaches were applied to datasets from African, Asian, and European population. In the first approach, orphan reads from all three populations were combined, and assembled using Velvet. This approach was likely to detect micSeqs that are present across all three populations. In the second approach, orphan reads from each population were assembled separately to detect the population specific micSeqs, this approach was intended to more easily identify sequences present in a single population.

### Functionality of micSeqs

To investigate the potential functions and gene annotations of the identified micSeqs, we identified RefSeq genes associated with micSeq anchor points (including 5Kb regions up and downstream) from RefSeq database downloaded from UCSC genome browser. We further investigated the expression profiles and TF-binding potential of micSeqs by mapping reads from RNA-seq and ChIP-seq data from previous studies [18–24, 27]. The short reads files are downloaded from SRA database of NCBI, and bowtie2 is used to map to micSeqs [46]. IGV is used to visualize the BAM files [48].

### PCR and Sanger sequencing

All PCR primers were designed by the Primer3 software, and can be found in Additional file 1: Table S1, S2. Five ng of genomic DNA was mixed with 5 unit of Platinum tag DNA polymerase and dNTP mixture in a 10 μl reaction. PCRs were carried out in a GeneAmp PCR System 9700 (Applied Biosystems). PCR products were resolved in 3% argarose gels. DNA bands were excised and purified using Qiagen Gel Extraction kits. The purified DNA fragments were sequenced by automated Sanger sequencing.

### Availability of supporting data

The sequence of 309 micSeqs in fasta format can be found in the supplementary section (Additional file 3), and an online database has been developed to store sequences, the predicted locations, neighboring genes, supporting evidence and other information, and can be accessed in http://proteomics.case.edu/micseqdb.

## Electronic supplementary material

Additional file 1: Table S1: PCR Primers for validation of 15 micSeqs. 15 micSeqs were randomly selected for experimental validation using PCR on an additional 38 samples from individuals with African (12 samples), Asian (12 samples) and European (14 samples) ancestry. The following table lists the primers designed using BatchPrimer3 (v1.0) program. The targeted micSeqs are also listed for each pair of primers. **Table S2.** Data Resource used in this study. **Figure S1.** PCR results for micSeq11710. PCR results for micSeq11710. Note that three bands are selected for Sanger sequencing, and one band doesn’t contain micSeqs (the lower bands). **Figure S2.** The sequence alignment of micSeq11710 and the sequences from Sanger sequencing for three bands from **Figure S1.** The presence and absence of micSeq11710 are confirmed. **Figure S3.** The sequence alignment of micSeq3 and BF687531.1 from dbEST. Note that it is the reverse complementary sequences of micSeq3 that shares high similarity with BF687531.1, indicating its opposite strand is expressed. (DOCX 277 KB)

Additional file 2: **TFbindingMicSeqs.xlsx summarizes the “** ***transcription factor/ChIP-seq samples*** **” for three micSeqs.** (XLSX 14 KB)

Additional file 3: **micSeq_aftMask.fa contains sequences for 309 micSeqs in fasta format.** (ZIP 27 KB)

## Abbreviations

micSeq: Missing common sequence
EBV: Epstein-barr virus
OEA: One-end anchor
NGS: Next generation sequencing
TF: Transcription factor.
