# Supplementary material for: Discovery of common sequences absent in the human reference genome using pooled samples from next generation sequencing
Source: BMC Genomics. 2014 Aug 16;15(1):685. doi: 10.1186/1471-2164-15-685 (PMC4148959; doi:10.1186/1471-2164-15-685)
Supplement: Supplementary file 1 — Additional file 1: Table S1: PCR Primers for validation of 15 micSeqs. 15 micSeqs were randomly selected for experimental validation using PCR on an additional 38 samples from individuals with African (12 samples), Asian (12 samples) and European (14 samples) ancestry. The following table lists the primers designed using BatchPrimer3 (v1.0) program. The targeted micSeqs are also listed for each pair of primers. Table S2. Data Resource used in this study. Figure S1. PCR results for micSeq11710. PCR results for micSeq11710. Note that three bands are selected for Sanger sequencing, and one band doesn’t contain micSeqs (the lower bands). Figure S2. The sequence alignment of micSeq11710 and the sequences from Sanger sequencing for three bands from Figure S1. The presence and absence of micSeq11710 are confirmed. Figure S3. The sequence alignment of micSeq3 and BF687531.1 from dbEST. Note that it is the reverse complementary sequences of micSeq3 that shares high similarity with BF687531.1, indicating its opposite strand is expressed. (DOCX 277 KB) [file 12864_2014_6377_MOESM1_ESM.docx]

**Discovery of Common Sequences Absent in the Human Reference Genome Using Pooled Samples from Next Generation Sequencing**

Yu Liu^1*^, Mehmet Koyutürk^1,2,3^, Sean Maxwell^1^, Min Xiang^4,8^, Martina Veigl^3,6^,

Richard S. Cooper^9^, Bamidele Tayo^9^, Li Li^3,5^, Thomas LaFramboise^3,4^,

Zhenghe Wang^3,4^, Xiaofeng Zhu^7^, Mark R Chance^1,3,4,*^

**Supplemental Materials**

**Supplemental Table 1**. Primers for validation of 15 micSeqs.

15 micSeqs were randomly selected for experimental validation using PCR on an additional 38 samples from individuals with African (12 samples), Asian (12 samples) and European (14 samples) ancestry. The following table lists the primers designed using BatchPrimer3 (v1.0) program. The targeted micSeqs are also listed for each pair of primers.

| **micSeqID** | **Primer(Forward)** | **Primer(Reverse)** |
| --- | --- | --- |
| micSeq11220 | TGGTCTGTGTTTGTTTGGCTA | GAAAAAGGGCTCAAATCAGAAA |
| micSeq11710 | AAGCCGTGTGCTCTGAAGAT | CCAGCCTTTCTTGTCAGTGC |
| micSeq13298 | TGGCCATTAAGGAATCTTCG | CTCACCTTTGTGCCTTACCC |
| micSeq143 | TCTACAGATCCAGATGCCAATG | TGATTTCAGAATTCCCTGTTGTT |
| micSeq16601 | CCCAGGAAAGCTTGTAATCG | AAATCAGTCATCCTCAGCCATT |
| micSeq1824 | GGAACTTCTTTTTAATCGGTGA | TCAGGCAATCACCACTGTTC |
| micSeq18764 | GAAGCCACAGGCACATCAC | GCCTCTTAAGCTCCTGCATC |
| micSeq2373 | TTTTGAGGGCTAGGCTTGAG | TTTTTGGAGGGAACGATGTC |
| micSeq2382 | AGGGGGAGATTTGAGTAGGG | TATGAACCATCCGCTCTCAG |
| micSeq28 | TCCTTTGCTAATCCCCACAC | ATTGTGACAAGGCAGCACAG |
| micSeq30 | CTTCAAGCAAGTGATCAGCA | AGACCTCTTCCAGTGCTTGG |
| micSeq349 | TGGCTCTATTTAGCCATAAGACA | CCTGCCTCCTCCTACAGATG |
| micSeq634 | GAGGGCAATCAACCTCTTTG | TATTTGAACCCTGGGCTTTG |
| micSeq71 | TGGACTTGAGACAGTGTGTGAATA | GTTCAAGCGAAAGGAAAAGC |
| micSeq9196 | CCCTCAAACTTGGTTAGGAAA | TCTCTCTCTCTCCCTCTCTCCA |

**Supplemental Table 2**. Data Resource used in this study.

| Data type | Resources | Purpose |
| --- | --- | --- |
| DNAseq | 1K Genome Project | Construction of micSeq |
| RNAseq | Ref 18 - 24 | Expression evidence |
| ChIPseq | ENCODE project | Identify micSeqs that can bind TF |
| Other primate genomes | UCSC genome browser | Validate micSeqs |
| Individual genomes | Ref 12, 16, and 17 | Validate micSeqs |

**Supplemental Figure 1**. PCR results for micSeq11710. Note that three bands are selected for Sanger sequencing, and one band doesn’t contain micSeqs (the lower bands)


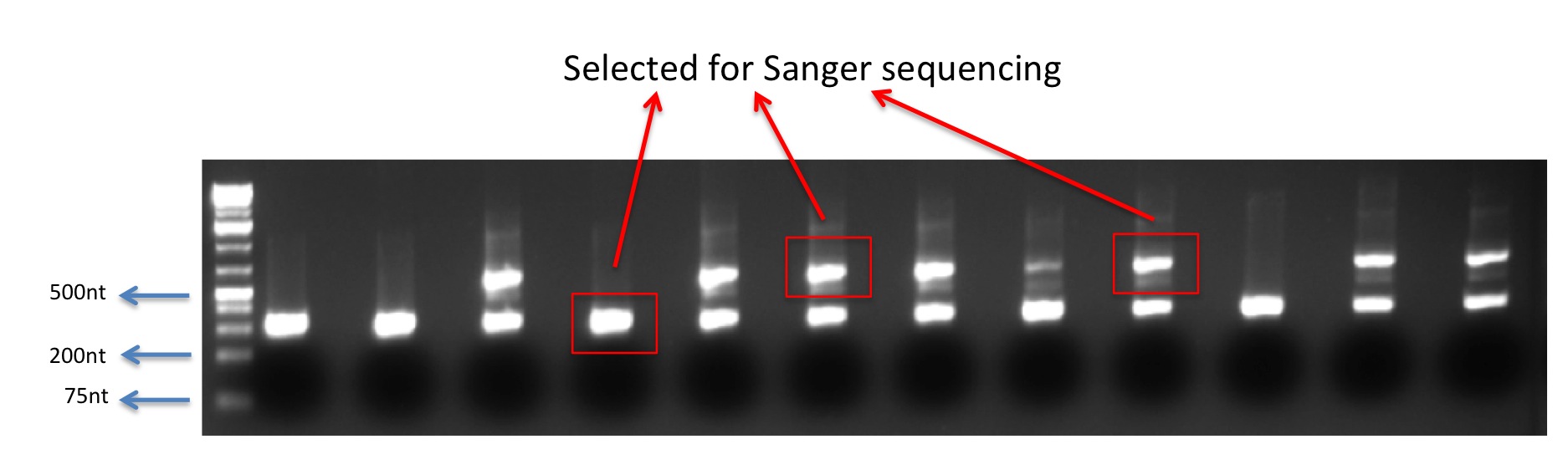


**Supplemental Figure 2.** The sequence alignment of micSeq11710 and the sequences from Sanger sequencing for three bands from Supplemental Figure 1. The presence and absence of micSeq11710 are confirmed.

**Supplemental Figure 2** (Continued).

**Supplemental Figure 3**. The sequence alignment of micSeq3 and BF687531.1 from dbEST. Note that it is the reverse complementary sequences of micSeq3 that shares high similarity with BF687531.1, indicating its opposite strand is expressed.
